# Going beyond still images to improve input variance resilience in multi-stream vision understanding models

**DOI:** 10.1038/s41598-024-66346-w

**Published:** 2024-07-04

**Authors:** Amir Hosein Fadaei, Mohammad-Reza A. Dehaqani

**Affiliations:** 1 College of Engineering, School of Electrical and Computer Engineering, University of Tehran, Tehran, Iran; 2 School of Cognitive Sciences, Institute for Research in Fundamental Sciences (IPM), Tehran, Iran

**Keywords:** Vision understanding, Vision classification, Spatiotemporal features, Deep neural networks, Network models, Neuroscience, Computational neuroscience, Visual system

## Abstract

Traditionally, vision models have predominantly relied on spatial features extracted from static images, deviating from the continuous stream of spatiotemporal features processed by the brain in natural vision. While numerous video-understanding models have emerged, incorporating videos into image-understanding models with spatiotemporal features has been limited. Drawing inspiration from natural vision, which exhibits remarkable resilience to input changes, our research focuses on the development of a brain-inspired model for vision understanding trained with videos. Our findings demonstrate that models that train on videos instead of still images and include temporal features become more resilient to various alternations on input media.

## Introduction

Our brains intricately process visual information in the form of a continuous spatiotemporal stream of visual input. The natural visual system employs visual footage or a combination of fused images to construct comprehensive representations of the surroundings, facilitating their classification and recognition. This visual data encapsulates both the spatial characteristics of objects and details within our environment, as well as temporal features that depict the relationships among spatial features over time and because of that, it shows resilience to spatial and temporal changes. The natural vision unfolds through two parallel pathways: the dorsal and ventral streams, both originating in the occipital lobe^1^. The occipital lobe, particularly the early visual cortex (V1-V4), processes fundamental visual elements such as angles and basic shapes. Additionally, it includes significant portions of both the ventral and dorsal visual streams, which extend toward other brain regions. Subsequently, the dorsal stream extends towards the parietal lobe, contributing significantly to our comprehension of positionings and motion, and the ventral pathway courses towards the temporal lobe, engaging in semantic analysis and object detection^2–4^. This intricate process underscores the dynamic interplay of spatial and temporal aspects in constructing our visual understanding^5,6^.

The evolution of video understanding algorithms aims to emulate visual cognition more closely in the human brain^7^. The emulation of natural vision stands as a central objective in both artificial intelligence and cognitive neuroscience. Video understanding models derive significant inspiration from the principles inherent in natural vision^8^. It is noteworthy that our innate visual capabilities are honed by exposure to spatiotemporal visual input, even influencing the processing of data derived from static images.

One remarkable quality of natural vision that researchers strive to replicate in models is its resilience against diverse input variations, culminating in the creation of invariant environmental representations. These environmental changes can manifest in various spatial or temporal alterations. Spatial changes encompass modifications in object appearance or shape, such as variations in lighting, brightness, rotation, translations, presence of obstacles, shifts in colors, or any noise or distortion that may impact each frame. Natural vision excels in object detection and comprehending their relevance across different environmental contexts. Notably, it demonstrates a remarkable ability to navigate through spatial changes, showcasing adaptability to alterations in the visual landscape. This adaptability proves crucial in real-world scenarios where environmental conditions are subject to constant flux. Moreover, natural vision’s robustness extends to temporal shifts introduced by factors like changes in perspective, camera angles, and shifts in time, which are prevalent in web and edited videos. Despite these variations, natural vision remains resilient, consistently perceiving the relationships between the data presented in different frames and can plan future actions and movements based on it^9^. This resilience underscores the aspiration to instill comparable adaptability and robustness in artificial models, contributing to their effectiveness in navigating dynamic and unpredictable visual environments.

Despite the introduction of various models and significant progress in achieving accuracy within more confined test environments, vision understanding models continue to face challenges in the presence of diverse spatiotemporal variances. Our hypothesis suggests that a significant contributing factor to this limitation lies in the extensive training of models with static information, leading to the loss of crucial temporal features and relationships. The removal of temporal features and information detailing how entities evolve can significantly diminish the efficiency of our models in detecting similar inputs that have undergone different alterations before being presented to the model. This temporal myopia, resulting from an overemphasis on static data during training, hinders the model’s ability to generalize and adapt to the dynamic nature of real-world scenarios. To address this limitation, it becomes imperative to reevaluate training methodologies, placing a greater emphasis on preserving and leveraging temporal information. The shift toward acknowledging and leveraging temporal data in video understanding models holds promising implications for enhancing the depth and accuracy of models in comprehending dynamic visual content^10,11^. Incorporating a more comprehensive representation of spatiotemporal relationships in the training process holds promise for enhancing the adaptability and robustness of vision understanding models across a broader spectrum of real-world scenarios.

The development of invariant representations plays a pivotal role in vision understanding tasks, intricately connected to the concept of generalization in our models. As previously mentioned, natural learning involves the observation and understanding of changes, similarities, and patterns, a process best facilitated through the analysis of videos rather than static images. However, the importance of temporal features, intimately linked to motion and alterations, has not been extensively explored in the training of vision models.

Our work contributes on two fronts. First, we introduce a simple multi-stream model inspired by the natural vision, tailored for image and video understanding, and compare its potential to other similar prominent video understanding models. The model, with its three streams comprising spatial, spatiotemporal, and audio elements, shows promising results in integrating temporal and audio features into multi-stream understanding models. Additionally, the proposed model is designed to enable training on both image and video understanding datasets and allows for independent experimentation with each stream. Second, we explore the model’s resilience to common input variations using modified images and videos, drawing comparisons with models trained solely on images. This research demonstrates the role of temporal features in creating invariant representations for image and video understanding.

## Method

In our exploration of the impact of transitioning to spatiotemporal features for creating invariant representations, we implemented a simple multi-stream model based on the existing multi-stream networks. Inspired by the principles of natural vision, the initial introduction of two-stream networks featured separate spatial and temporal channels^12–14^. However, these networks faced limitations in scaling for longer videos. To address this challenge, the Temporal Segment^15^ and Deep Temporal Linear Encoding (TLE)^16^ networks proposed a segmentation approach, dividing videos into smaller sections to enable aggregation and encoding of the final result for temporal fusion. In earlier iterations, two-stream models utilized intermediary media as inputs for the temporal stream. However, more recent models^17^ have introduced novel methods for extracting temporal features. Drawing inspiration from the pathways of natural vision, the Slowfast Network^18^ employs two pathways for the extraction of spatiotemporal features. One pathway, known as the Fast stream, provides higher temporal coverage with a high frame rate input, allowing for the extraction of finely tuned temporal-focused spatiotemporal features. The other pathway, referred to as the Slow stream, offers lower temporal coverage with a lower frame rate input, providing a robust foundation for extracting features rich in spatial information. Employing multiple parallel pathways extends beyond spatial and temporal streams of vision. By integrating a mixture of expert classifiers^19^, we can incorporate audio information into our network, thus influencing the classification outcomes.

These models feature a dedicated temporal stream alongside a pre-trained image understanding model (spatial stream). The temporal stream is designed to emulate the dorsal pathway observed in natural vision, mirroring the way our brains process temporal information^20^. Concurrently, the pre-existing image understanding channel corresponds to the ventral pathway, focusing on spatial aspects. This dual-stream configuration essentially transforms the model from a single-stream image understanding model to a more robust two-stream video understanding model, aligning seamlessly with the primary objective of this research.

The spatial stream in our implementation comprises a pre-trained ResNet-101^21,22^, renowned for its proficiency in object detection and image classification tasks. In addition, the second stream employs the fusion of multiple sampled frames to generate a more sophisticated representation and a long-term feature repository for the video dataset^23,24^. This feature repository equips the network with the capability to infer relationships between various entities. Within the temporal channel, a fusion layer is implemented, utilizing a slow fusion model^25^. This stream also includes a fully connected layer and a NetVLAD^26^ layer to enhance the results following fusion.

We include a third-stream channel to train the network using audio data. The audio stream incorporates fully connected, and NetVLAD layers^26^, mirroring the structure of the temporal stream. In this implemented model, the spatial stream is ResNet and the temporal and audio streams are similar to the streams used in the WILLOW model^27^. The outputs from these three distinct streams result in vectors of the same size of $c \times 1$, where c is the number of classes, denoted as $V_{S}$, $V_{T}$, and $V_{A}$. Employing a Mixture of Expert Classifier in conjunction with context gating, we combine these three vectors using weights $g_{S}$, $g_{T}$, and $g_{A}$ from the same size of $c \times 1$. The Mixture of Expert Classifier vector is defined as follows:

1 $$\bf{V} = \bf{V_{S}} .\bf{g_{S}} + \bf{V_{T}} . \bf{g_{T}} + \bf{V_{A}} .\bf{g_{A}} .$$

The weights $g_{S}$, $g_{T}$, and $g_{A}$ are both trainable and adaptable, contingent upon the input, as determined by the Mixture of Experts Classifier model. They enable the model to flexibly adjust its reliance on each stream according to the contextual information. Put simply, the mixture of experts classifier combines the outputs of the three streams using learnable weights, providing insight into the model’s confidence in each stream’s classification capability for every class. Additional details regarding these weights are provided in the results section, where we explore the effectiveness of each stream. This adjustment is followed by a context gating mechanism and a classifier layer applied to the output vector generated by combining the results from the various streams.

**Figure 1:**
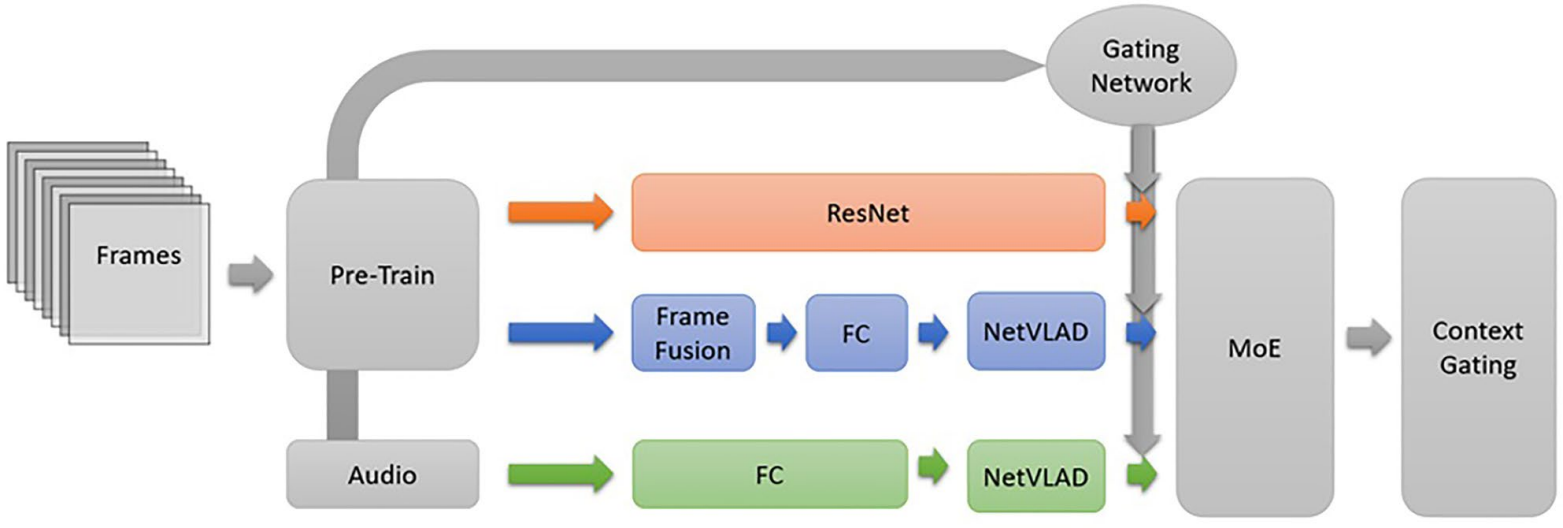
The model consists of three streams: a spatial stream (depicted in red) takes a single-image input and utilizes a pre-trained ResNet^21,22^ for spatial feature detection. The spatiotemporal stream (illustrated in blue) conducts slow fusion on multiple frames to extract spatiotemporal features from the input video. The third stream is an audio stream (depicted in green), contributing significantly to the model’s understanding of the input data. The combined output is then processed by the Mixture of Experts classifier and fine-tuned using attention-based context gating.

Context gating functions as an attention-based filter, allowing the network to modulate its confidence in output classes. For instance, in a video featuring skiing, the model may exhibit high confidence in detecting trees. However, if these trees are in the background and hold less relevance, the network can adjust its output confidence accordingly using a self-attention filter. The overarching model schema is visualized in (Fig. 1).

### Datasets and evaluation metrics

To perform this experiment, various datasets for image and video understanding were employed. For pretraining the image understanding stream and assessing the model’s resistance to modified input images, the ImageNet dataset^28^ was utilized. Renowned for its extensive coverage, ImageNet^28^ is a widely recognized dataset for image understanding and object recognition, comprising over 14 million images across 1000 classes.

To train the model comprehensively on video understanding data, the Youtube-8M^29^ dataset was employed, boasting a vast collection of over 6 million videos spanning 3862 classes. Recognized as one of the largest and most extensively validated video understanding datasets, Youtube-8M was chosen as the training set for this model. For assessing the impact of manipulated input videos in video understanding tasks, the Holistic Video Understanding (HVU)^30^ dataset, derived from videos in YouTube-8M^29^, was utilized. HVU^30^ contains over 572,000 videos and 3142 classes.

For this study, we generated modified datasets derived from ImageNet^28^ and HVU^30^ to assess the model’s performance in image and video understanding tasks. These modified datasets underwent random spatial alterations, allowing us to evaluate the model’s resilience to such changes. In each experiment, we employed five modified versions of the datasets and tested each of these datasets and the unaltered version with a five-fold cross-examination approach. In all instances of training the three-stream model, including the pretraining with YouTube-8M for subsequent experiments outlined in this research, no alterations were applied to the YouTube-8M data. Notably, with the HVU dataset^30^, derived from the same source as YouTube-8M^29^, we meticulously verified video uniqueness using their respective YouTube IDs to prevent any test leakage, ensuring that the pretraining data does not include the test videos in each instance. Further details on these modified datasets, along with the results of the model’s performance on each, are presented in the subsequent section.

To evaluate the model, various metrics, including accuracy, global average precision (GAP)^31^, and mean average precision (mAP), were employed. The model generates up to 20 pairs $\left( {e_{v, k} , f_{v,k} } \right), k \le 20$; where $e$ represents a class label, and $f$ indicates the confidence level for the predicted class label. Global average precision (GAP)^31^ is defined as follows:

2 $$GAP = \mathop \sum \limits_{i = 1}^{10000} P\left( i \right) \left[ {R\left( {i - 1} \right) - R\left( i \right)} \right] .$$

Here, $P\left( i \right)$ represents the total number of correct labels with confidence above the threshold, divided by the total number of correct classes for all videos at rank $i$. Similarly, $R\left( i \right)$ denotes the total number of correct labels with confidence above the threshold, divided by the total number of classes in all videos at rank $i$. The mentioned threshold is dynamically updated based on rank and is calculated as $\tau_{i} = \frac{i}{10000}$.

mAP@K, which stands for mean average precision ranked at K, is calculated similarly to GAP. It involves computing the average precision for each class, considering the maximum rank of K, and essentially applying the GAP calculation for each class individually.

3 $$AP_{c} @K = \mathop \sum \limits_{i = 1}^{K} P_{c} \left( i \right) \left[ {R_{c} \left( {i - 1} \right) - R_{c} \left( i \right)} \right].$$

Secondly, we calculate mAP by averaging the resulting average precision (AP) for each class:

4 $$mAP@K = \frac{1}{C}\mathop \sum \limits_{i = 1}^{C} AP_{c} @K,$$

where $C$ represents the total count of classes in the dataset.

## Results

### Training and evaluation of three stream model

**Figure 2:**
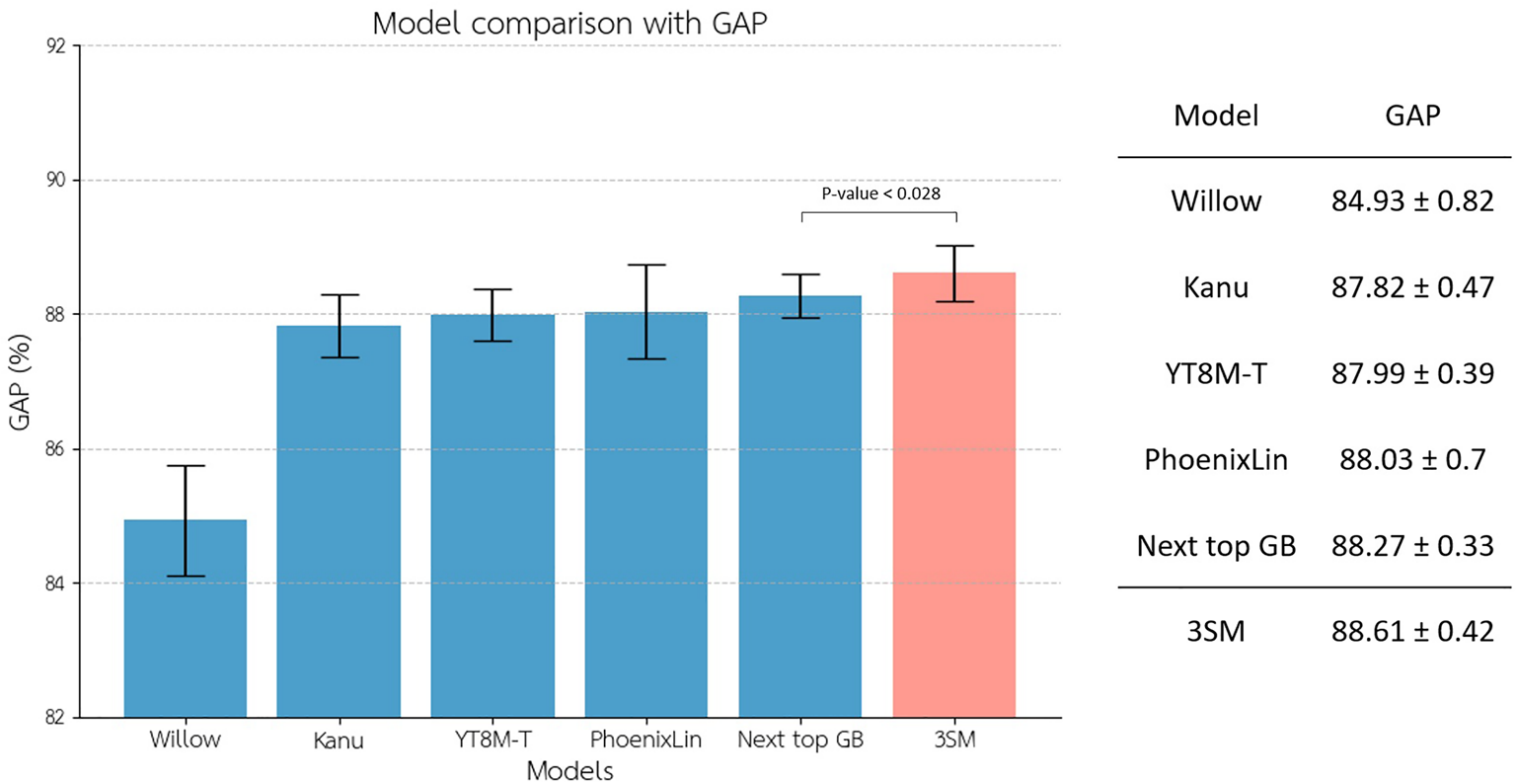
The performance of the implemented three-stream model (3SM) with audio, spatial, and temporal channels was compared to top-performing models from the YouTube-8M competitions using Global Average Precision (GAP)^31^ as the evaluation metric. The objective is to establish the model’s relevance, not to assert its status as a state-of-the-art model in video understanding, as that is not the goal of the research. The error bars show 3*STD for each model.

We conducted training on the network using the YouTube-8M dataset^29^, where all videos were trimmed to a duration of 10 s, equivalent to 300 frames (or less in some cases, depending on the frame ratio of the original data). The spatial network takes the central frame (frame 150) as input, while the temporal network incorporates 30 input frames at a 10:1 sampling ratio. Selecting the central frame was aimed at capturing a representative snapshot of the video content, under the assumption that the central frames will mostly contain the majority of the assigned classes. The model was trained with an adaptive learning rate. Our results, applying a fivefold cross-validation, demonstrate enhanced performance (*p*-value < 0.028, Bootstrap test, *N* = 100,000) in video understanding tasks in comparison to other models that participated in the YouTube-8M competitions, as presented in (Fig. 2). The models compared with the 3SM in this study include WILLOW^27^, the winner of the first video understanding YouTube-8M challenge^29^, and the highest-performing models from the second YouTube-8M challenge^31^, such as Kanu^32^, YT8M-T^33^, PhoenixLin^34^, and Next Top GB^35^. These models employ similar structures for feature augmentation in video understanding, providing a robust baseline for evaluating our model’s general performance on the YouTube-8M dataset classification.

**Figure 3:**
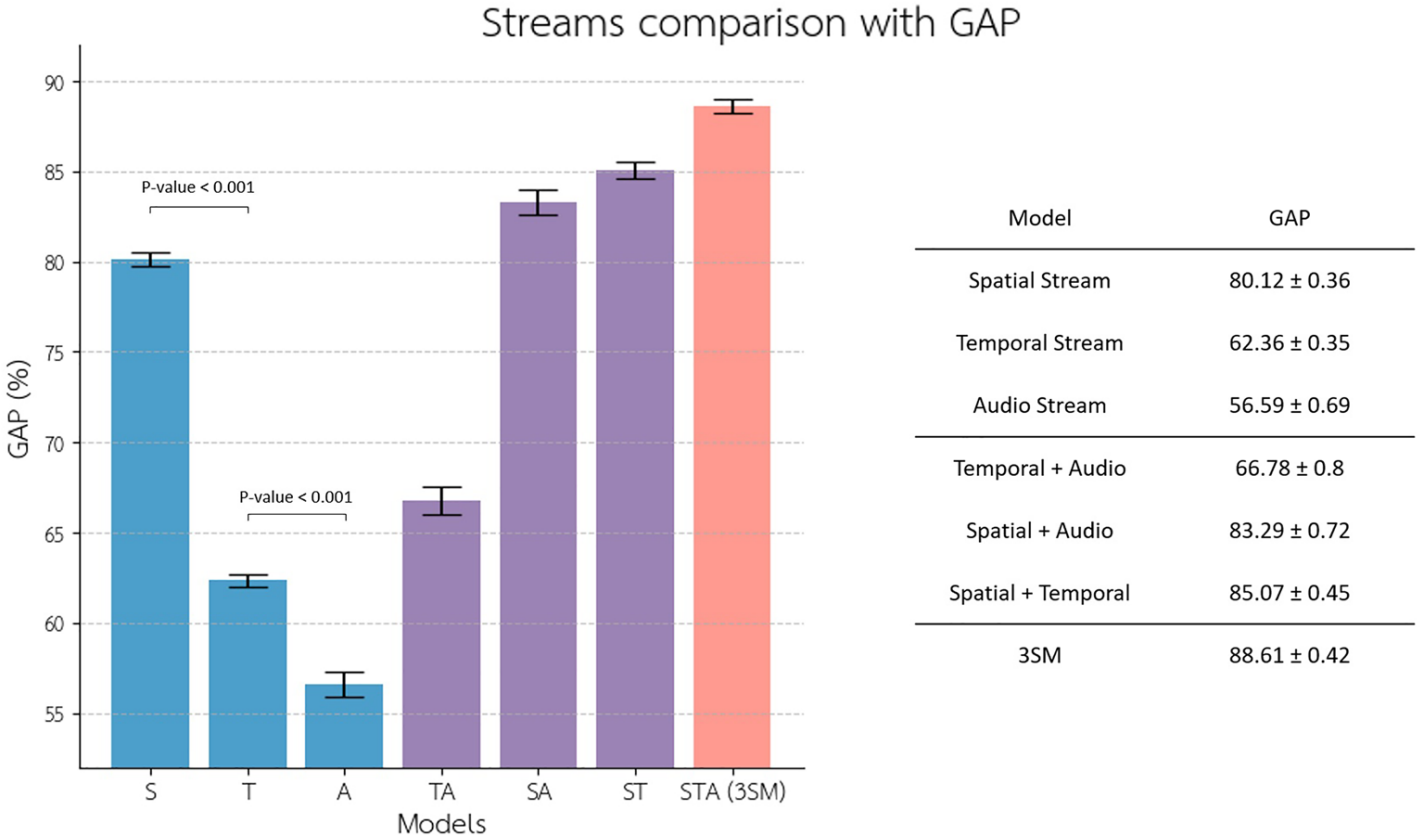
We examined the contribution of each stream in the three-stream model. The performance of the implemented three-stream model (3SM) is compared to each of its streams (Spatial, Temporal, and Audio streams) both independently and when one stream is excluded. The blue bars represent the GAP when only a single stream is present, while the purple bars depict the GAP when only a single stream is excluded. The letters S, T, and A stand for Spatial, Spatiotemporal, and Audio and represent their related included streams.

In addition, we examined the contribution of each stream initially by assessing the accuracy of each stream individually and then by selectively excluding certain streams from the model. It’s important to note that we did not retrain the model for each of these results. All the findings are derived from the same trained models, where certain streams were excluded from the mixture of experts only during the testing phase (Fig. 3). The results were obtained through fivefold cross-validation. Among the streams, the one with the most parameters (the spatial stream) exhibits the highest significance, followed by the temporal and audio streams, in that order (*p*-value < 0.001, Bootstrap test, *N* = 100,000).

**Figure 4:**
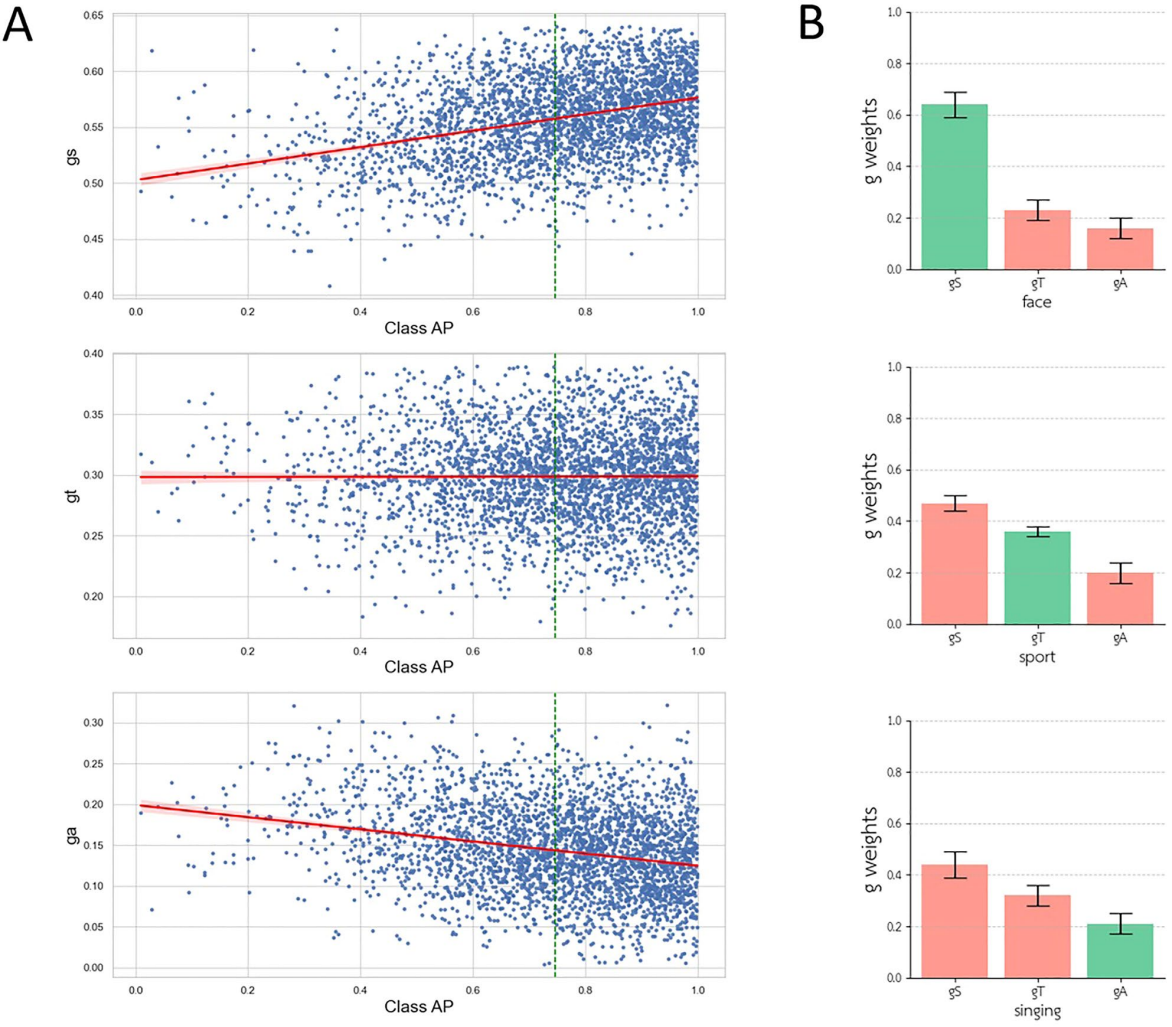
We examined the contribution of each stream in the three-stream model. **(A)** We generated regression plots to analyze the relationship between the weights (${\text{g}}_{{\text{S}}}$, ${\text{g}}_{{\text{T}}}$, and ${\text{g}}_{{\text{A}}}$) and the AP for each class. The spatial, temporal, and audio streams approximately exhibited regression slopes of 0.073, 0.001, and -0.074, respectively. **(B)** We analyzed the mixture of experts classifier weights for each stream concerning three classes: face, sport, and singing. These classes exhibited some of the highest weights for one of the streams, which are represented in green for each class. The error bars show 3*STD for each model/parameter.

Secondly, we delved into the examination of the trained weights of the mixture of experts for each class to assess the model’s confidence in utilizing the resulting vector generated by each stream when calculating its final output. The trained weights $g_{S}$, $g_{T}$, and $g_{A}$ serve as valuable metrics for analyzing the relevance of each stream to each classification task. However, it’s important to note that these results demonstrate how our model was trained to rely on each of its streams for each class and do not directly indicate the accuracy of each stream’s result or the final result for that class. The comparison of the distribution of these weights with the average precision (AP) achieved for each class reveals that the spatial stream was more significant for classes with higher AP, whereas the audio stream was the least significant contributor to class AP. Additionally, as a quality check, we reported these weights for three classes: “face,” “sport,” and “singing.” These sampled classes exhibited high AP and a significant result for one of the three stream weights after excluding outlier data. As expected, the class with greater expected motion and temporal information showed a higher $g_{T}$, and the class with valuable audio information demonstrated a higher $g_{A}$ (Fig. 4).

### Image understanding model

To evaluate the influence of the temporal stream and determine whether the three-stream model creates an invariant representation when the temporal stream is incorporated versus when it is not, we conducted separate experiments using image understanding and video understanding datasets. For the experiment with the image understanding dataset, we first trained the model on the YouTube-8M dataset, as explained in the previous section, to pretrain the model streams. Subsequently, we exclude its audio stream due to the absence of audio data in the ImageNet and image understanding datasets. Additionally, we employ a single-stream ResNet, mirroring the number of parameters in the spatial stream of the three-stream model, for comparative analysis. We began by utilizing the ImageNet dataset^28^ and subsequently generated modified data from its images. These modifications encompassed several aspects by applying the following probabilities:

- 15% probability of reducing image brightness.
- 15% probability of rotating the image 45 degrees clockwise.
- 15% probability of scaling up the image and selecting the central area across all scales.
- 55% probability of keeping the image unchanged.

**Figure 5:**
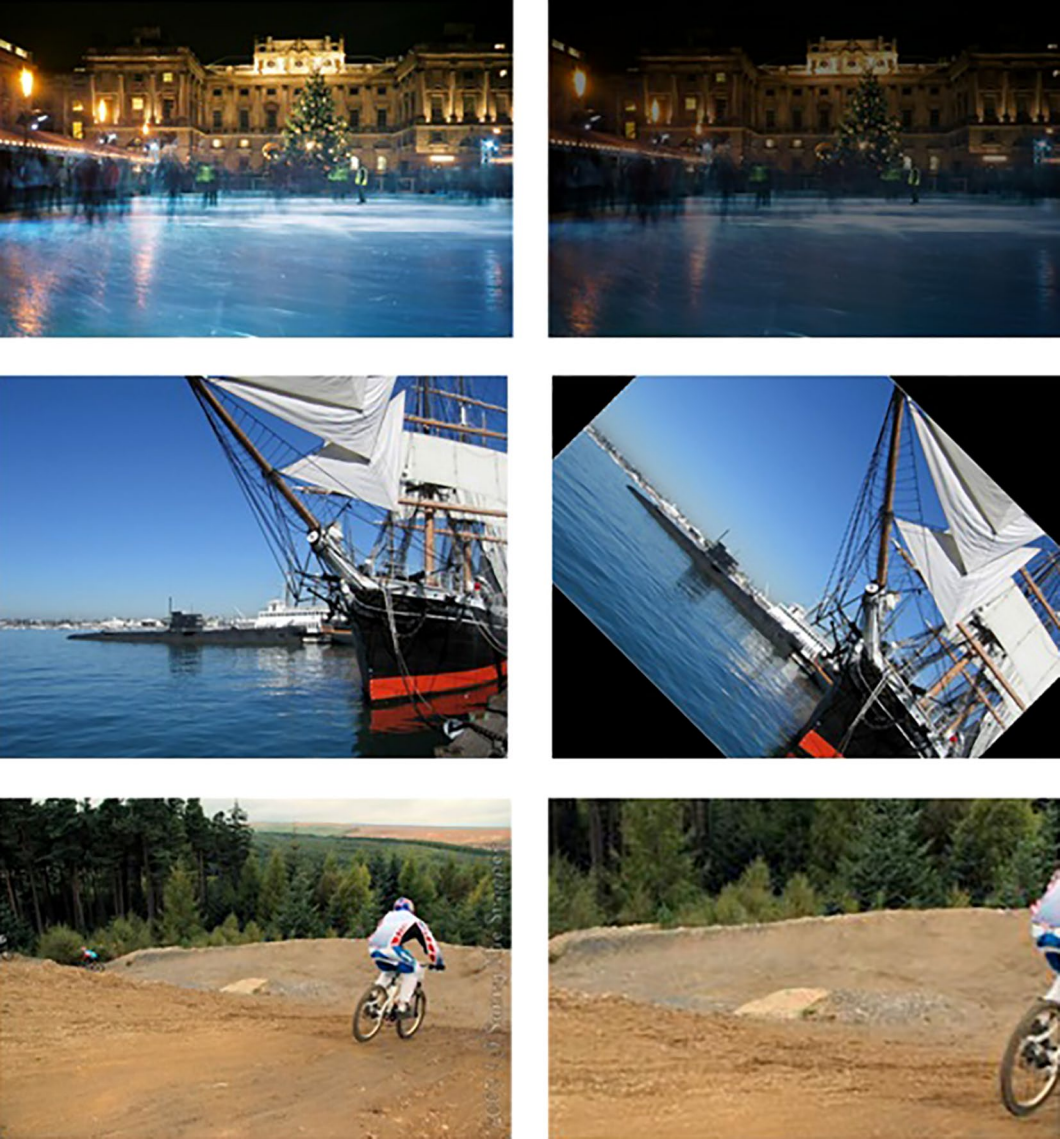
Here are some examples of modifications applied to the ImageNet dataset, with the modified pictures displayed on the right side. These samples were correctly interpreted by the 2-stream model, whereas the single-stream model struggled to understand them after the modifications.

These three modifications address some of the basic spatial alterations. While we trained the two-stream model using videos, our evaluation involved both the ImageNet dataset and the modified ImageNet dataset^28^. For the temporal stream, we used 30 repeated frames of each image as input. Samples of both original and modified images are illustrated in (Fig. 5).

**Figure 6:**
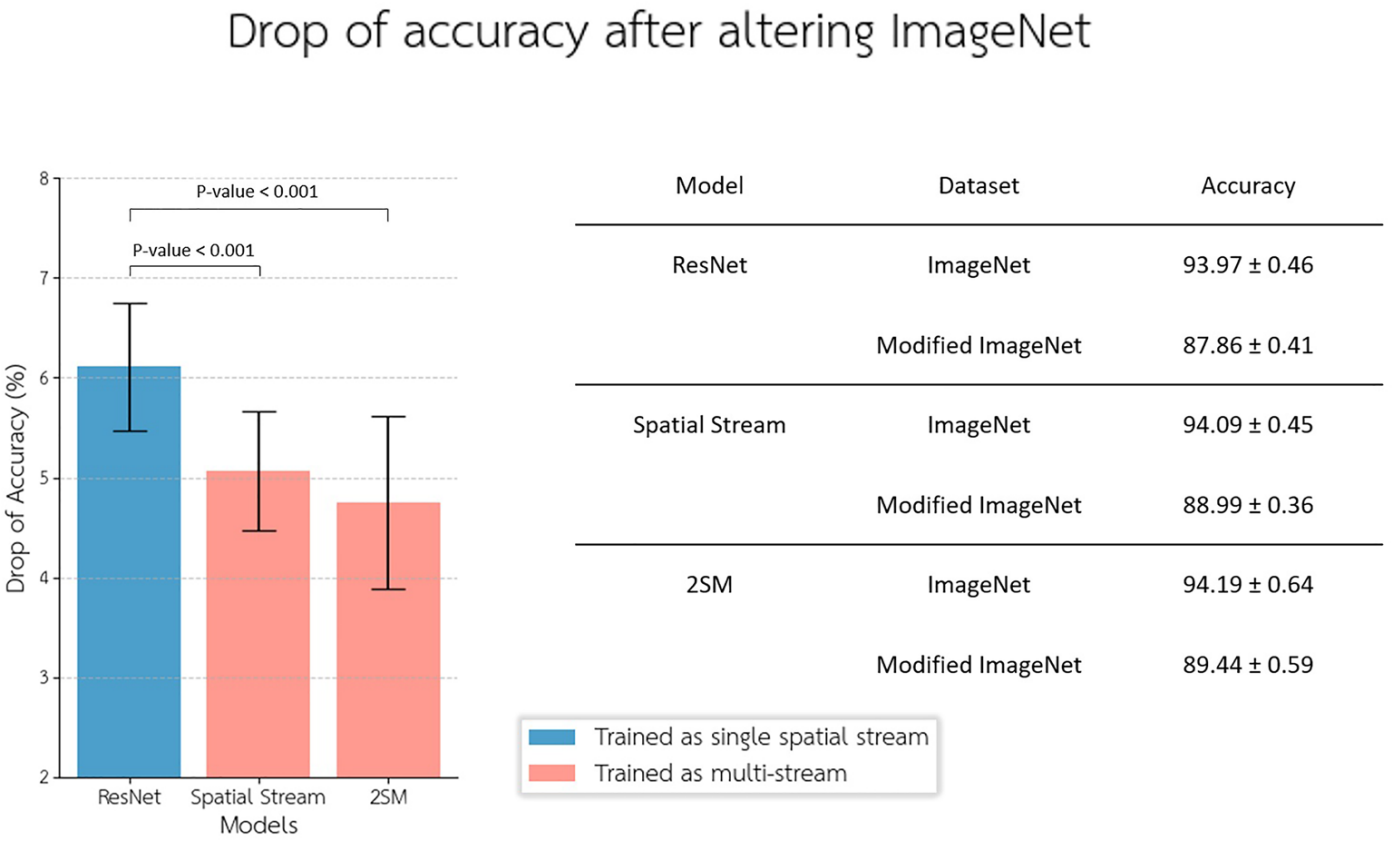
This plot presents a comparison of the drop rate of accuracy between the two-stream model, its spatial stream, and the single-stream model (ResNet). We conducted evaluations using both unaltered and modified image understanding datasets (ImageNet). The findings indicate a notable improvement in accuracy retention when both streams are included during training, compared to the spatial stream-only model. The error bars show 3*STD for each model.

This dataset lacks temporal dimension information as it consists of a single repeated image over time. However, this test can demonstrate how a model trained for video understanding, equipped with a dedicated stream for extracting spatiotemporal features, can enhance invariant representations for image understanding tasks. The results indicate that the fine-tuned two-stream model does not surpass the single-stream model in terms of accuracy for image understanding tasks significantly on ImageNet dataset (*p*-value > 0.201, Bootstrap test, *N* = 100,000) as shown in (Fig. 6). Nevertheless, it is noteworthy that the single-stream model experienced a statistically significant drop in accuracy compared to the two-stream model (*p*-value < 0.001, Bootstrap test, *N* = 100,000) and its spatial stream (*p*-value < 0.001, Bootstrap test, *N* = 100,000). For this experiment, we utilized five randomly generated versions of the modified ImageNet dataset. The drop rate of accuracy was calculated using a fivefold cross-validation approach and alongside all these versions.

### Video understanding model

Next, we employed the HVU^30^ video understanding dataset, which comprises over 9 million training labels, 3142 classes, and more than 572 thousand videos (Some of the videos were not available anymore on YouTube at the time of this research). For this test, we introduced modifications to the HVU video dataset, including the following alterations:

- 10% probability of adding a yellow color filter.
- 10% probability of adding a red color filter.
- 10% probability of reducing video brightness.
- 10% probability of rotating the video 45 degrees counterclockwise.
- 60% probability of keeping the video unchanged.

**Figure 7:**
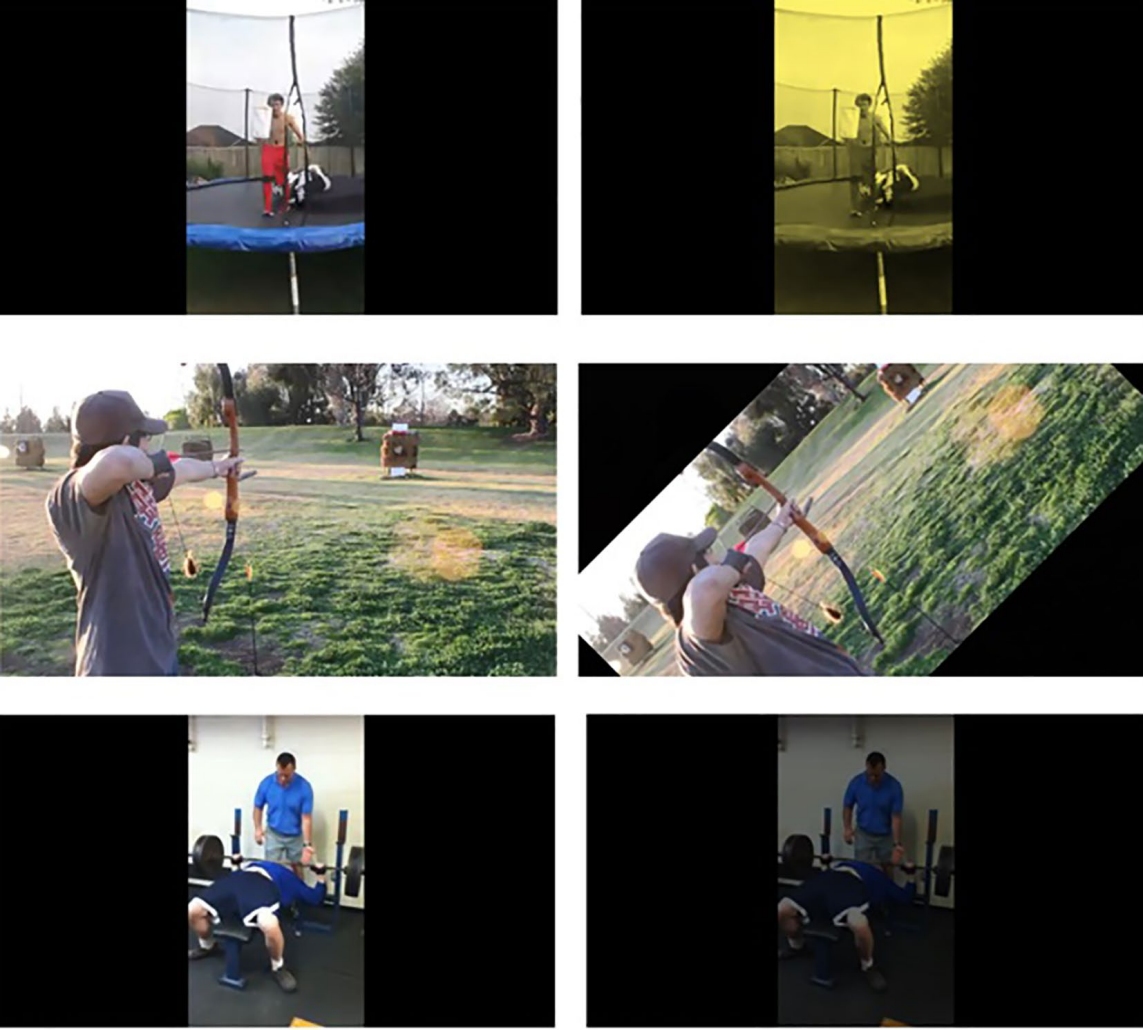
Here’s the HVU dataset along with some examples of the modifications we applied to it, with the modified videos displayed on the right side. These samples were correctly interpreted by the 3-Stream model, whereas the single-stream model struggled to understand them after the modifications.

**Figure 8:**
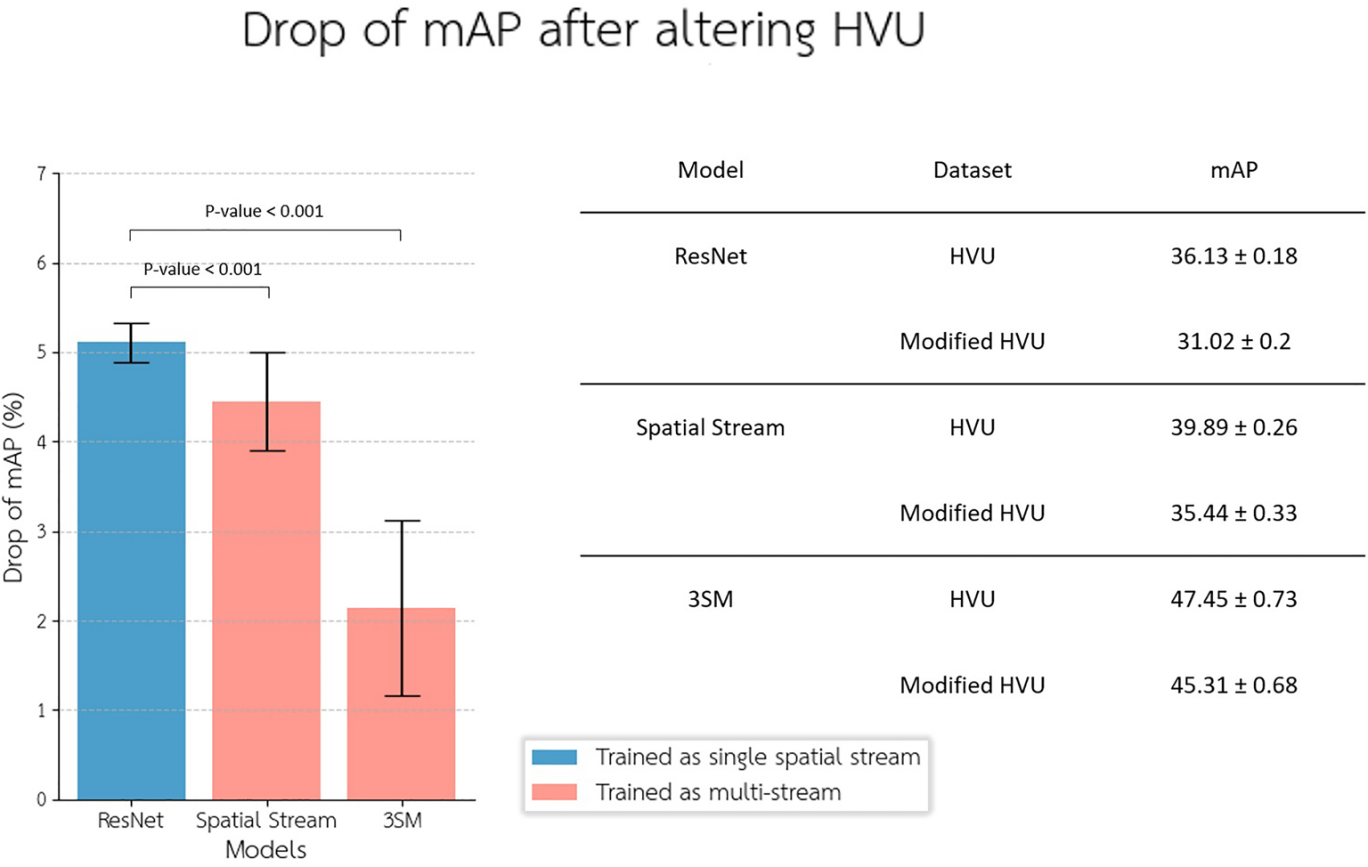
This table presents a comparison of the drop of mAP (mean average precision) between the two-stream model, the spatial stream, and the single-stream model (ResNet). The evaluations were conducted using both unaltered and modified video understanding datasets. The outcomes reveal a significant reduction in the mAP drop when incorporating the spatiotemporal stream, compared to using only the single-stream model. The error bars show 3*STD for each model.

Sample frames from the altered video dataset can be viewed in (Fig. 7):

We utilized the central frame of the videos for the spatial stream, and we selected 30 frames for the temporal stream. The results indicate an improvement in mAP (mean average precision)^29^ when the temporal stream and features are included in the training phase (*p*-value < 0.001, Bootstrap test, *N* = 100,000). Additionally, there is a notable decrease in mAP when only the spatial stream is used during training compared to the three-stream model with the modified dataset (*p*-value < 0.001, Bootstrap test, *N* = 100,000). The spatial stream, which is part of the three-stream model, also demonstrates greater resilience in the drop of mAP compared to the ResNet model (*p*-value < 0.001, Bootstrap test, *N* = 100,000), as shown in (Fig. 8). For this experiment, we employed five randomly generated versions of the modified HVU dataset. The drop rate of mAP was calculated using a fivefold cross-validation approach.

**Figure 9:**
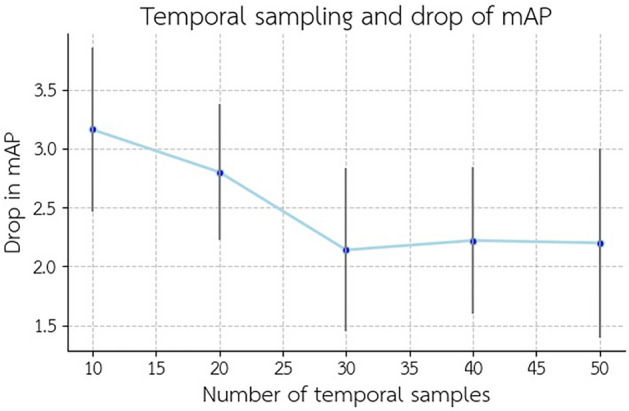
Increasing temporal sampling does lead to changes in the mAP drop rate. However, it’s essential to note that augmenting temporal sampling may not necessarily result in an improved resistance of the model to input variance. The model exhibited the least decline in mAP when using a temporal sampling rate of 30 frames. The error bars show 3*STD for each experiment.

In our previous experiments, we employed 30 frames for temporal sampling and fusion out of a total of 300 frames. To further investigate the impact of temporal sampling, we conducted more experiments with fivefold cross-validation with varying temporal sampling rates. (Fig. 9) illustrates how the model was influenced by these different temporal sampling rates. The data indicates two key points. First, the temporal stream significantly contributes to the reduction in the drop of mAP when facing augmented input, as altering this stream’s input while keeping the rest of the structure unchanged can impact this outcome. Secondly, selecting the appropriate number of frames for temporal sampling, based on the temporal stream structure and the data, can influence the model’s resilience to input variance changes.

## Discussion

We observe with a continuous spatiotemporal stream of visual input rather than still images. Natural vision exhibits a remarkable tolerance for visual variance. Drawing inspiration from this observation and the results obtained, we can assert that transitioning toward brain-inspired video understanding models with multi-stream spatiotemporal feature extraction will enhance the model’s resilience to various input changes. Models focused on spatiotemporal feature extraction and analysis, trained on video datasets, develop a deeper understanding of spatial and temporal variations, thus enabling better generalization for both image and video understanding tasks.

The principal aim of this study is to investigate the challenge of input invariance by contrasting single-stream spatial models with multi-stream spatiotemporal models. In this context, our implementation is exclusively compared to other models that have been previously assessed using the Youtube-8M dataset^29^, which allows for such comparisons. Transformer models and even more advanced multi-stream models like the Slowfast Network^18^ have the potential to attain superior results in video understanding tasks. However, these structures make it less straightforward to observe and assess the transition from spatial features to spatiotemporal features and their influence on input variance.

Incorporating a second stream for temporal feature extraction leads to the creation of a long-term feature bank, essentially forming a comprehensive impression of the entire video. This ‘general impression’ proves invaluable in identifying long-term dependencies among spatial features over the temporal domain. Moreover, it enables the model to discern patterns and variations within the spatial domain as they evolve. By including a temporal stream, we enhance the model’s robustness against input changes. Much like the dorsal pathway in natural vision, which facilitates understanding of how objects relate to each other and the observer’s body in space, the temporal stream within a two-stream model can represent the overarching context of spatial features and their temporal interrelationships.

The results unequivocally demonstrate that training our models with video data enhances their resilience to input variances both in video understanding and in image understanding tasks. Even in cases where the image lacks inherent temporal information, the model can derive substantial benefits from the spatiotemporal structure and the temporal stream. This enables the model to grasp dynamic changes and construct invariant representations, contributing to improved performance in image comprehension.

It’s noteworthy that our model didn’t necessarily outperform the ResNet model in accurately discerning and classifying unmodified visual data when confronted with an image-understanding dataset. However, our model exhibited a significant reduction in the drop rate of accuracy when encountering input spatial changes compared to the single image stream, as illustrated in the results section. This validates its practical utility, particularly in environments where understanding models may be deployed and face various spatial changes that are not necessarily categorized or predictable. Additionally, given the remarkable tolerance of our natural vision for spatial input changes, our modeling approach can serve as a valuable tool in investigating the source of this resilience. It’s also worth noting that our model demonstrated a significant improvement in classifying unmodified data when confronted with video-understanding datasets compared to the single spatial stream.

In terms of model complexity and resource requirements during training, it’s worth noting that the utilization of the mixture of experts classifier allows for parallel training of each stream. While this accelerates training speed, it can pose challenges for less powerful hardware to handle the computational load of three neural networks simultaneously. During parallel training, the slowest stream becomes the bottleneck for the overall training process. In our setup, the spatial stream is the slowest, with over 48 million trainable parameters. The temporal stream comprises 24 convolutional slow fusion, the fully connected layer, and NextVlad layer^34^, totaling approximately 17 million trainable parameters. Conversely, the audio stream consists of approximately 10 million parameters, making it the smallest stream in the current implementation.

Multi-stream networks, while not currently at the forefront of video understanding models, have served as valuable tools in our research. Our use of two-stream models allowed us to make meaningful comparisons between image-understanding and video-understanding models for a range of tasks, leveraging their simplicity and structural advantages. Multi-stream models, essentially extensions of image understanding models with temporal fusion, provided an excellent foundation for our research, enabling us to explore the effectiveness of spatiotemporal models in creating invariant representations. The proposed multi-stream model draws significant inspiration from the WILLOW architecture^27^ for context gating and the Mixture of Experts structure for combining classification results. This design enables training on both image and video understanding datasets, while also facilitating independent experimentation with each stream, aligning with the primary objective of this research. In developing this model, we opted to exclude PCA (Principal component analysis) and all preprocessing steps utilized in the WILLOW structure^27^ and other models in YouTube-8M competitions^29,31^.

Furthermore, we incorporated an audio stream into the multi-stream model, although it does not directly affect the subsequent tests on the spatial stream analyzing model resilience based on the training data type. Nevertheless, this addition has the potential to enhance the overall model performance. We detailed the impacts of each stream and their potential enhancements to the model in the Results section.

There are several avenues for future research in this field. Exploring the inclusion of an Audio stream and audio data to enhance the creation of invariant representations further. The inclusion of audio and the auditory network is a crucial aspect of natural vision that often complements visual understanding. These sensory inputs work in tandem, enriching the overall perception after undergoing separate processing through parallel pathways. Our model is designed with a three-stream structure, incorporating an audio stream. Drawing inspiration from the principles of natural vision, we hypothesize that audio plays a role in constructing invariant representations of objects and events within the environment. It’s worth noting that further dedicated research will be required to delve deeper into this intriguing topic.

While we acknowledge the effects of certain architectures, structures, and specific training steps, such as applying transformations with pretraining data, on increasing the model’s resilience against input spatial changes, the primary focus of this research was to analyze the effects of training with temporal features on the model’s resilience against these changes. Therefore, any additional steps applied to the model that couldn’t be replicated for the solo ResNet model in comparison could compromise the validity of the results due to confounding factors. Implementing such steps to further enhance the model’s resilience in parallel could be the subject of future studies.

It is important to acknowledge the scope of the experiments and the resultant findings. These experiments were conducted based on predetermined types of spatial modifications applied to the input datasets. While these modifications encompassed various types of spatial changes aimed at enhancing generalization results, there is merit in examining the effects of each spatial change individually. Future research could focus on assessing the model’s resilience to each modification separately, offering deeper insights into its adaptability and robustness. Throughout these experiments, two types of modifications were introduced to the training datasets. Additionally, each dataset underwent the experiment with the alternative modification type, with the results documented in the supplementary information.

Our model serves as a foundational structure for comparing and assessing the resilience of different models and architectures to various spatial input alterations. A potential future direction for research involves conducting similar experiments and comparing various other established image-understanding models, especially the ones that are also inspired by human recognition^36–40^ and other video-understanding models such as the Temporal Segment Network^15^ and Temporal Shift Module^41^, within this framework. Looking ahead, it’s crucial to recognize the potential of transformer models with multi-head self-attention, which have attained state-of-the-art results in video understanding^42^ These models have exhibited exceptional performance in achieving generalized understanding and forming invariant representations across various visual tasks. Vision Transformer (ViT)^43^ in image understanding and Multiscale Vision Transformers (MViT)^44^, TimeSFormer^45^, and Video Vision Transformer (ViViT)^46^ for Video understanding, are some examples of the transformer-based models for visual understanding. We also need to mention LVLM transformer models such as Video-LLaVA^47^ and agent-based LLM models such as DoraemonGPT^48^, which have shown great results in understanding dynamic scenes and processing multimedia inputs. Therefore, one avenue for future research lies in investigating transformer models and their adaptability to input changes, which could shed further light on their capabilities in the context of spatiotemporal modeling.

Additionally, investigating the potential benefits of incorporating feedback mechanisms between spatiotemporal components and layered-wise model comparisons is a subject to consider. Lastly, delving deeper into the alignment between models and the principles of natural vision presents an intriguing area for future investigations.

In summary, our exploration into the realm of spatiotemporal feature extraction has allowed us to cultivate a more generalized understanding and establish invariant representations in our models. This, in turn, equips the model with greater resistance to input variances and changes. To empirically assess this theory, we conducted experiments involving a single-stream spatial model and a two-stream model trained on video data, featuring a dedicated temporal stream. The findings from these experiments validate our hypothesis: the model employing multi-stream spatiotemporal feature extraction exhibits a smaller decline in accuracy when confronted with modified image or video datasets (by more than 3% in mAP for Video understanding tasks). Thus, our results underscore the advantage of training models with video data, even in scenarios where temporal information offers less additional insight for understanding static images.

Our research underscores the importance of designing models and training understanding tasks with videos for achieving better generalization. In essence, our model has demonstrated the ability to create invariant representations applicable to both image and video understanding tasks.

### Supplementary Information

Supplementary Information.

## Data Availability

The related model files and tools for this experiment are publicly available at https://github.com/amfad33/3-stream-model. The original datasets, YouTube-8M^29^ (https://research.google.com/youtube8m/), ImageNet^28^ (https://www.image-net.org/index.php), and HVU^30^ (https://holistic-video-understanding.github.io/), have been previously published. Details on reconstructing the models used for training and conducting experiments on these datasets, along with information on all random modifications applied to them, are thoroughly described in this article. The modifications were introduced randomly in multiple trials to ensure the statistical significance of the findings.
