# Supplementary material for: Going beyond still images to improve input variance resilience in multi-stream vision understanding models
Source: Sci Rep. 2024 Jul 4;14:15366. doi: 10.1038/s41598-024-66346-w (PMC11224316; doi:10.1038/s41598-024-66346-w)
Supplement: Supplementary file 1 — Supplementary Information. [file 41598_2024_66346_MOESM1_ESM.docx]

Supplementary Information

Amir Hosein Fadaei ^1^, Mohammad-Reza A. Dehaqani ^1 2 *^

[hosein.fadaei@ut.ac.ir](mailto:hosein.fadaei@ut.ac.ir), [dehaqani@ut.ac.ir](mailto:dehaqani@ut.ac.ir)

1- School of Electrical and Computer Engineering, College of Engineering, University of Tehran, Tehran, Iran

2- School of Cognitive Sciences, Institute for Research in Fundamental Sciences (IPM), Tehran, Iran

* Corresponding Author

Supplementary information for the paper “Going beyond still images to improve input variance resilience in multi-stream vision understanding models”

Submitted to Nature Scientific Reports

**Analyzing the modifications applied to datasets**

During these experiments, we introduced two types of modifications to our training datasets. The first type of modification, which we will call Type One, was originally applied to the ImageNet dataset to assess the impact of training the model with videos on image understanding datasets. These modifications were randomly applied with the following probabilities:

1. 15% probability of reducing image brightness.
2. 15% probability of rotating the image 45 degrees clockwise.
3. 15% probability of scaling up the image and selecting the central area across all scales.
4. 55% probability of keeping the image unchanged.

The second type of modification, referred to as Type Two, was initially implemented on the HVU video understanding dataset. These modifications were likewise randomly applied, with the following probabilities:

1. 10% probability of adding a yellow color filter.
2. 10% probability of adding a red color filter.
3. 10% probability of reducing video brightness.
4. 10% probability of rotating the video 45 degrees counterclockwise.
5. 60% probability of keeping the video unchanged.


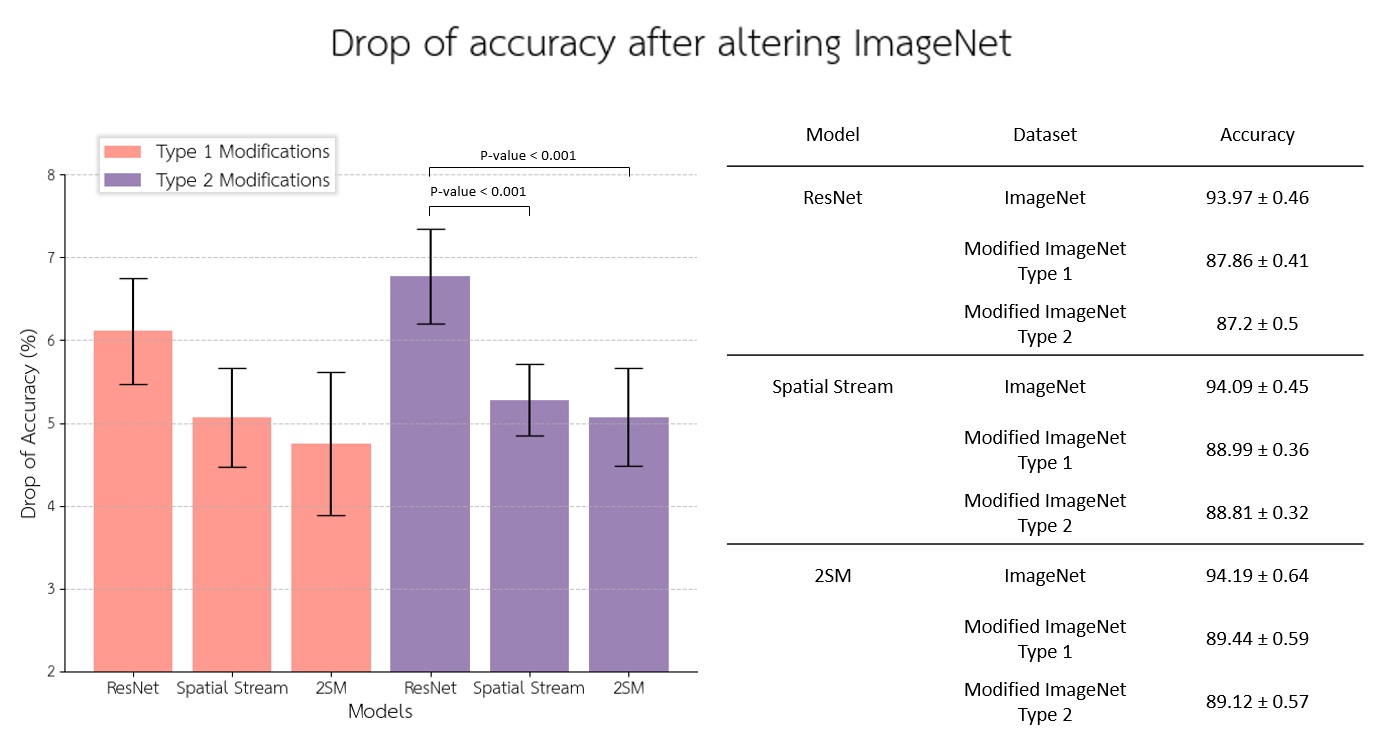


*Figure S1. We replicated the experiment conducted on the ImageNet dataset, focusing on image understanding models, using the modifications originally applied to the HVU dataset. In the presented results, the red bars illustrate the accuracy drop observed when employing Type 1 modifications (previously applied to ImageNet and reported in the paper), while the purple bars represent this drop rate when utilizing Type 2 modifications (originally applied to HVU). These findings substantiate our assertions that the applied modifications were sufficiently diverse for generalization, and altering them within a reasonable spectrum still yields comparable conclusions in this experiment.* *We performed 5-fold cross-validation over the 5 modified versions of each dataset, and statistical significance was determined using bootstrap tests (N = 100000). The error bars show 3*STD for each model.*


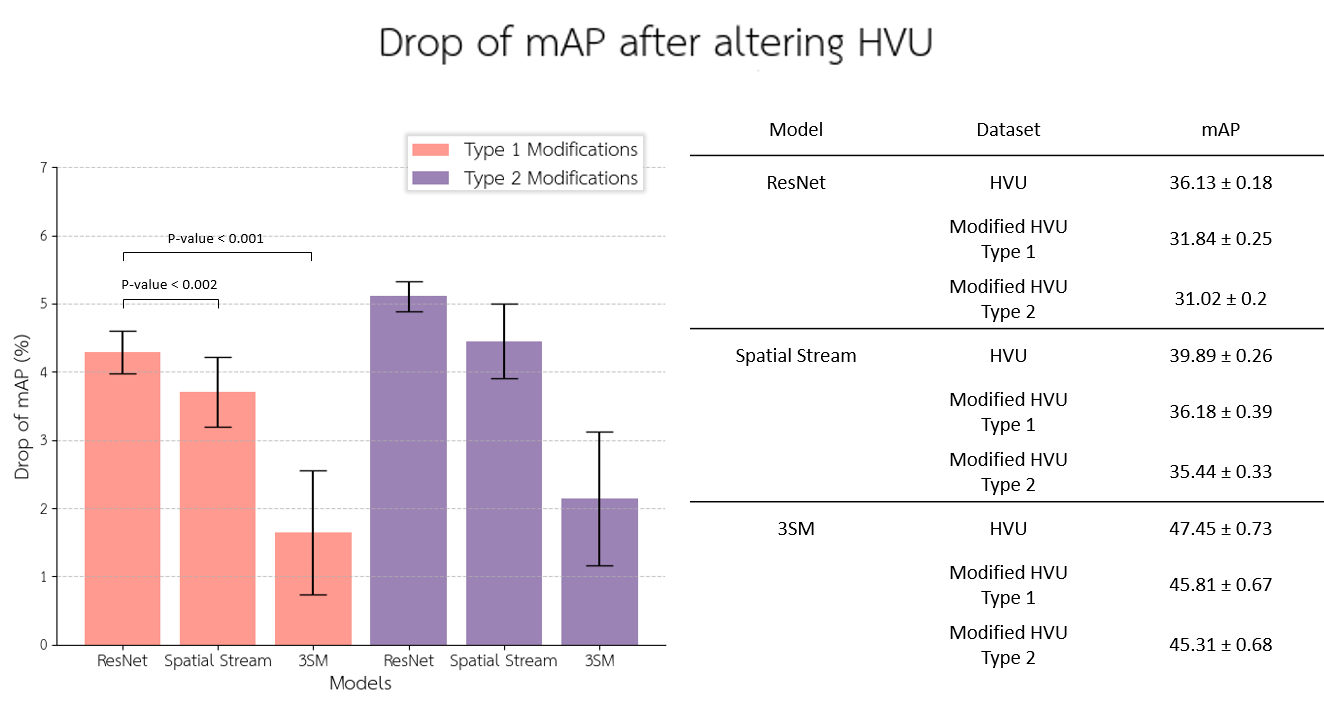


*Figure S2. We replicated the experiment conducted on the HVU dataset, focusing on video understanding models, using the modifications originally applied to the HVU dataset. In the presented results, the red bars illustrate the drop in mAP (mean Average Precision) observed when employing Type 1 modifications (previously applied to ImageNet), while the purple bars represent this drop rate when utilizing Type 2 modifications (originally applied to HVU and reported in the paper). These findings reinforce our assertions that the applied modifications were sufficiently diverse for generalization, and altering them within a reasonable spectrum will still yield similar conclusions in this experiment. We performed 5-fold cross-validation over the 5 modified versions of each dataset, and statistical significance was determined using bootstrap tests (N = 100000). The error bars show 3*STD for each model.*
